# Treating lower limb muscle cramps: a Cochrane systematic review

**DOI:** 10.1186/1757-1146-4-S1-O17

**Published:** 2011-05-20

**Authors:** Fiona Hawke, Kate Walter, Vivienne Chuter, Joshua Burns

**Affiliations:** 1 Discipline of Paediatrics and Child Health, The University of Sydney, Westmead, NSW, 2145, Australia; 2 Podiatry Program, The University of Newcastle, Ourimbah, 2258, Australia; 3 Institute for Neuroscience and Muscle Research, The Children’s Hospital at Westmead, Sydney, NSW, 2145, Australia

## Background

Muscle cramps affect approximately 1 in 3 people in the general community each year. Many interventions are available for lower limb cramps, but not all are efficacious or supported by evidence. Many treatments are controversial, no treatment guidelines exist, and many people experience no benefit from the interventions prescribed. In clinical trials, no drug treatment for cramps has demonstrated consistent effectiveness and safety and none are approved by the Australian Pharmaceutical Benefits Scheme or the American Food and Drug administration for the most common form of cramp; nocturnal leg cramps. Due to the unclear risk/benefit ratio of many drug treatments, patients are encouraged to try non-drug treatments. The aim of this project was to systematically review the evidence for non-drug treatments for lower limb cramp.

## Methods

We searched MEDLINE, EMBASE and CENTRAL up to April 2010 for randomised controlled trials of any type of non-drug treatment for lower limb cramp in adults and children. Review methods were according to a peer-reviewed Cochrane protocol.

## Results

1,284 potentially relevant trials were retrieved and screened for inclusion. Trials were translated from Swedish, French and German. Authors of five trials were requested to provide more information. Of these studies, four have been excluded and the inclusion of the final study is pending the provision of additional information by the author. Complete results will be presented at the conference.

## Conclusions

This systematic review identifies a desperate need for clinical research of non-drug therapies for lower limb muscle cramps. We are now investigating factors associated with lower limb muscle cramp to help identify non-drug therapeutic targets for sufferers of this common complaint.

